# Metagenomic Detection of Viruses in Aerosol Samples from Workers in Animal Slaughterhouses

**DOI:** 10.1371/journal.pone.0072226

**Published:** 2013-08-14

**Authors:** Richard J. Hall, Mily Leblanc-Maridor, Jing Wang, Xiaoyun Ren, Nicole E. Moore, Collin R. Brooks, Matthew Peacey, Jeroen Douwes, David J. McLean

**Affiliations:** 1 Institute of Environmental Science & Research, National Centre for Biosecurity & Infectious Disease, Wallaceville, Upper Hutt, New Zealand; 2 Centre for Public Health Research, Massey University, Wellington Campus, Wellington, New Zealand; The Pirbright Institute, United Kingdom

## Abstract

Published studies have shown that workers in animal slaughterhouses are at a higher risk of lung cancers as compared to the general population. No specific causal agents have been identified, and exposures to several chemicals have been examined and found to be unrelated. Evidence suggests a biological aetiology as the risk is highest for workers who are exposed to live animals or to biological material containing animal faeces, urine or blood. To investigate possible biological exposures in animal slaughterhouses, we used a metagenomic approach to characterise the profile of organisms present within an aerosol sample. An assessment of aerosol exposures for individual workers was achieved by the collection of personal samples that represent the inhalable fraction of dust/bioaerosol in workplace air in both cattle and sheep slaughterhouses. Two sets of nine personal aerosol samples were pooled for the cattle processing and sheep processing areas respectively, with a total of 332,677,346 sequence reads and 250,144,492 sequence reads of 85 bp in length produced for each. Eukaryotic genome sequence was found in both sampling locations, and bovine, ovine and human sequences were common. Sequences from WU polyomavirus and human papillomavirus 120 were detected in the metagenomic dataset from the cattle processing area, and these sequences were confirmed as being present in the original personal aerosol samples. This study presents the first metagenomic description of personal aerosol exposure and this methodology could be applied to a variety of environments. Also, the detection of two candidate viruses warrants further investigation in the setting of occupational exposures in animal slaughterhouses.

## Introduction

Significant excess risks of lung cancer [1]–[7] and haematologic neoplasms [8]–[10] have been observed in slaughterhouse workers in both cohort and case-control studies conducted in several countries including New Zealand. Although no specific causes have been identified, a biological cause is considered most likely [1] as the risk is highest in those parts of meat processing plants where workers are either exposed to live animals, directly involved in animal slaughter, or exposed to biological material contained in animal urine, faeces and blood. With the aim of identifying potential causes of excess risk for work-related cancer in slaughterhouse workers we have conducted an investigation of a range of plausible biological exposures in meat processing plants, including the collection of personal inhalable dust/bioaerosol samples for analysis using high-throughput sequencing (HTS; also known as deep sequencing, next-generation sequencing) to describe the metagenome in slaughterhouse air.

Technical developments in HTS mean that it is now both cost-effective and technically possible to determine the profile of all known organisms present within a sample by massively parallel analysis of DNA (or RNA) sequences present, a field now termed metagenomics [11], [12]. A diverse number of sample types within the biosphere have already been explored using these techniques, such as profiling the organisms present in indoor urban air [13], those within the ocean [14], the determination of genomic variation in the human gut microbiome [15] or the discovery of previously unidentified pathogens [16].

For bacteria, the 16S rRNA gene is conserved across all species yet provides enough sequence diversity to resolve the bacterial metagenome present within a sample, often down to the genus level [17], [18]. In contrast, the detection of viruses using metagenomics is confounded, as viruses do not share a common genetic element. To overcome this, virus discovery using HTS relies upon *de novo* unbiased sequencing datasets, and the subsequent comparison to all known sequences contained within the public databases using comparative search algorithms such as BLAST [19].

Before the availability of HTS, metagenomics relied upon small scale cloning of random DNA (or cDNA) obtained from a sample [20]. Detection of a virus is only likely if high concentrations of virus are present [11]. More sensitive techniques for virus detection are available, such as real-time PCR, but these tests are highly specific and require *a priori* knowledge of the type of virus expected to be within the sample and so are unsuitable for viral discovery applications.

The discovery of a novel arenavirus in a fatal disease cluster in 2008 represents one of the first applications of HTS metagenomics to resolve the aetiology of an acute disease in humans [21], and since this time many more novel viruses have been identified for other acute diseases [16]. HTS metagenomics also holds promise for the resolution of putative aetiological agents in chronic diseases such as multiple sclerosis [22] and various malignancies [23], [24], however, determining viral aetiology for chronic disease can be problematic, as the detection of a virus may be circumstantial or the time-course of disease is such that the virus is only present in the early stages before symptoms are manifest [16]. The selection of an appropriate sample type is also of critical importance. One aim of epidemiological studies is to establish whether there is a link between a pathogen being present in an environment and the occurrence of a disease. The first step toward determining the (biological) risk of disease faced by individuals when exposed to certain environments is to determine the profile of micro-organisms present in that environment. In this study we describe what we believe is the first unbiased metagenomic analysis of air samples that are representative of individuals’ personal exposure, with the aim of characterising potential biological risk factors in the occupational setting of an animal slaughterhouse.

## Materials and Methods

### Ethics Statement

Approval for this study was given by the New Zealand Multi-Region Ethics Committee, approval number MEC/10/08/083. Participants provided written informed consent after they had viewed an information sheet on the aims and requirements of the study. The consent procedure was approved by the ethics committee, and consent forms filed in order to document this process.

### Aerosol Sampling Method

Personal samples of the inhalable fraction of dust/bioaerosol in workplace air were collected during full shift periods in separate cattle and sheep slaughterhouses. Workers wore portable sampling pumps (Gilian 3500, Sensidyne Inc.) calibrated to an air flow of 2 L.min^−1^, fitted with PAS-6 sampling heads containing 1 µm pore size polytetrafluoroethylene (PTFE) filters (Millipore, Merck) attached in the breathing zone. On completion of sampling, filters were removed from the sampling heads using aseptic technique and placed in sterile petri dishes, sealed in zip-lock plastic bags and transported on dry ice to the laboratory where they were stored in a −80°C freezer until processed.

### Nucleic Acid Extraction

PTFE filters were removed from −80°C storage and rinsed with 400 µL of RT-PCR grade water (Ambion, AM9935) followed by addition of 800 µL of lysis buffer from the MagMAX Viral RNA Isolation kit (Ambion, AM1939), which also co-purifies DNA. Nucleic acid extraction was followed as per the manufacturer’s instructions except carrier RNA was not included. Each aerosol sample was eluted in 40 µL of RT-PCR grade water (Ambion, AM9935).

### High-throughput Sequencing

A minimum of 1 µg of DNA is required for the Illumina TruSeq™ DNA sample preparation kit V2 to generate libraries for sequencing. Individual aerosol samples provided less than 5 ng of DNA per sample, as determined by PicoGreen assay (Invitrogen, P11496). Amplification was performed to increase the yield of DNA, using multiple displacement amplification in the Illustra GenomiPhi V2 DNA amplification kit (GE Healthcare 25-6600-30) as per the manufacturer’s instructions. Nine individual samples from each of the cattle or sheep areas were pooled into one sample by combining 10 µL of DNA from each individual filter. The combined pooled DNA was then precipitated by addition of 100% ethanol and 3 mM sodium acetate with centrifugation at 17,000×*g* for 15 minutes. Pooled DNA samples were re-suspended in 5 µL of water and 1 µL was used in the multiple displacement amplification reaction.

For each of the two pooled samples more than 1 µg of DNA was produced and this was sequenced on an IlluminaHiSeq2000 instrument (New Zealand Genomics Limited, Otago Genomics Facility, University of Otago, Dunedin, New Zealand) using an Illumina TruSeq DNA library preparation.

### Bioinformatic Analysis

**Figure 1:**
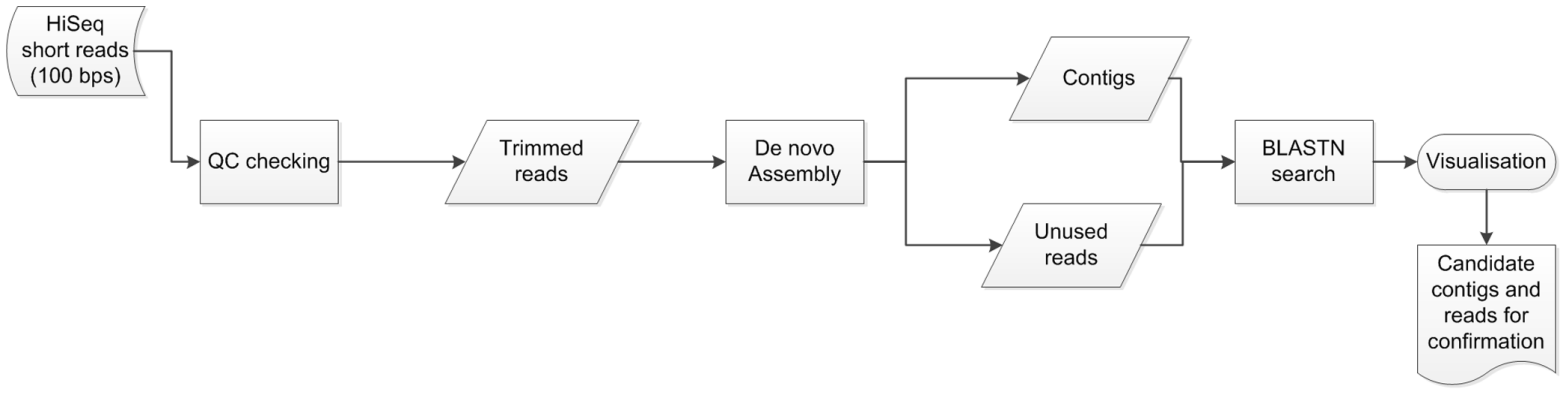
Bioinformatics pipeline. Approach used for the discovery of viral sequences in *de novo* metagenomic sequencing data from the Illumina HiSeq2000 dataset obtained from the personal aerosol samples.

The bioinformatics workflow/pipeline is depicted in Figure 1. FastQC was used to check the quality of the Illumina HiSeq2000 sequencing data. Based on the summary report from FastQC, the read length was trimmed to 85 bp after applying an average quality score threshold of 30, and duplicate reads were collapsed using FASTX-Toolkit (version 0.0.13). Velvet 1.2.07 [25] was used to conduct a *de novo* assembly of the cleaned Illumina HiSeq2000 data, the analysis was performed using default settings, except for the use of a kmer of 51, retaining the unassembled sequences and retaining a log file that records and matches the unique identifier of sequences used to build each contig. Assembled contigs were compared to the genbank non-redundant nucleotide sequence database (National Center for Biotechnology Information, Bethusda, MA, USA; downloaded June 2012) using BLASTN [26] and an e-value threshold for reporting of 0.0001. Unique reads were compared to a database of virus sequences derived from the complete genbank non-redundant nucleotide database with the same threshold. BLAST output was visualised in MEGAN (version 6.0) [27] for taxonomic classification, using a minimum score of 150, minimum support value of 5 and read complexity of 0.3. Mapping of vertebrate virus sequences to reference genomes was achieved using Bowtie 2 [28] and the default parameters for local mapping, where sequences were mapped to the complete reference genome JQ963485.1 for human papillomavirus type 120 isolate CL3805, and complete genome GU296398.1 for WU polyomavirus isolate T9. Sequence data of interest was extracted from the Illumina HiSeq2000 dataset in order to design the confirmatory PCR assays.

### Confirmatory PCR

Sequences identified by high-throughput sequencing were confirmed using a customised PCR assay for the target sequence of interest. Primers were designed directly from the sequencing data using Geneious 5.5.6 (Biomatters, New Zealand). A 50 µL PCR reaction was used containing 5 µL sample DNA, 44 µL Platinum® PCR SuperMix (Invitrogen), and 1 µL of each primer (10 nM concentration). The primer sets used consisted of BDNA-wupoly1-fwd (AGGGTTTCTGCCTTTTCTTTGGTG) and BDNA-wupoly1-rev (TGTTCACAGGCAACACCACCT) for WU polyomavirus, and BDNA-papvirus2-fwd (GCATCCCCAGAATGCTCACCCA) and BDNA-papvirus2-rev (TGGAGCACGTATTGGCTCCCG) for human papillomavirus 120. All PCR amplifications were performed using the following thermocycling profile: initial denaturation of 94°C for 2 minutes, then 40 cycles of 30 seconds at 94°C, 30 seconds at 53°C and 30 seconds at 68°C, followed by a final extension of 5 minutes at 68°C. Amplified DNA was visualised by agarose gel electrophoresis. Amplicons used in sequencing were purified using a Qiagen MinElute PCR Purification kit and underwent direct sequencing using the sanger method (Big Dye Terminator v.3.1 cycle sequencing kit, Applied Biosystems, Nieuwerkerk, NL) on a capillary sequencer (Model 3100 Avant, Applied Biosystems).

## Results

The amount of nucleic acid required for high-throughput sequencing prohibits the use of samples with a low yield of DNA, such as the requirement for 1 µg of DNA in the Illumina TruSeq DNA kit. For the aerosol samples obtained in this study, we pooled n = 9 personal aerosol samples from cattle and sheep processing areas respectively. In addition, a multiple displacement amplification method was used given that the starting amount of DNA extracted from each individual PTFE filter was less than 5 ng. We were able to successfully amplify over 1 µg of DNA for each of the two pooled samples which allowed for library preparation and sequencing on the Illumina HiSeq2000 platform (New Zealand Genomics Limited, Otago Genomics Facility, University of Otago). There were 332,677,346 sequence reads produced for the cattle-area pooled sample and 250,144,492 sequence reads produced for the sheep-area pooled sample, with a read length for both of 85 bp after quality-based trimming.

**Figure 2:**
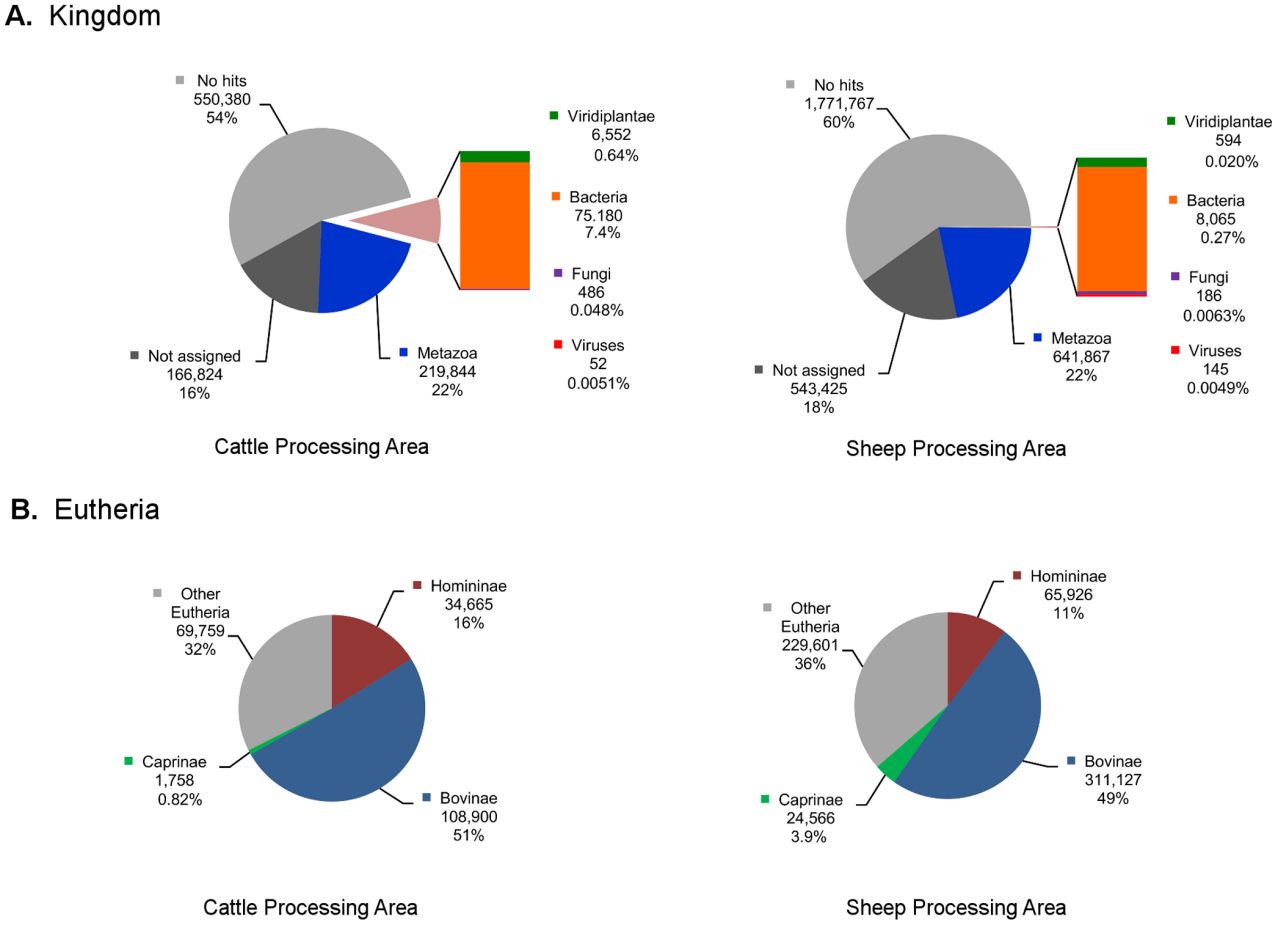
Contig sequence diversity in personal aerosol samples. Taxonomic assignment of BLASTN output using MEGAN for both the cattle processing area (left) and the sheep processing area (right). Kingdom level assignment is shown (A) and the proportions of contig assignments within the Eutheria branch are also shown (B). For Eutheria the proportions of Bovinae (includes cattle), Caprinae (includes sheep) and Homininae (includes human) sequences are shown, given that these were the relevant Eutheria branch organisms present within the animal slaughterhouse. BLASTN hits to all other Eutheria have been shown separately. The number of contigs assigned to each taxon is shown below the taxon name, and the percentage value is also shown (2 s.f.).

A BLASTN search of the assembled contigs from the cattle processing and sheep processing areas showed that the organisms present were consistent with the environment the sample was taken from (Figure 2), a full description of the taxonomic assignments for this dataset are available in Figure S1 and S2. Eukaryotic genome sequence was found in both sampling locations, and Bovinae (cattle), Caprinae (sheep) and Homininae sequences were common (Figure 2).

Bacteria and bacteriophage were also found to be present (Figure 2). Vertebrate viruses were identified in the cattle pooled sample (Table 1) for the assembled contigs with 8 significant hits in the BLASTN search recorded against human papillomavirus 120 (HPV120) and 6 significant hits for WU polyomavirus (WUPyV). There were no significant BLAST hits for the 85 bp sequences that were not assembled into contigs (data not shown), likely due to the short read length. The majority of viral sequences detected in the sheep-area pooled samples were retroviruses (Table 1). It was not possible to confirm whether these retroviral sequences were just endogenous retroviral sequence from the host genome of sheep, and so further investigation of these sequences was not pursued.

**Table 1:** Identity of virus contigs as reported by BLASTN search against the non-redundant genbank nucleotide database, and subsequent taxonomic assignment by MEGAN.

Viral Taxa	Number of contigs	
	Cattle Processing Area	Sheep Processing Area
**Retroviruses**	0	123
**WU polyomavirus**	6	0
**Human papillomavirus 120**	8	0
**Bacteriophage**	38	22
**Po-circo-like viruses**	2	0
**Total**	54	145

Consistent with previous metagenomic datasets, a large proportion of the sequences in the cattle and sheep processing areas (Figure 2) could not be assigned to any taxa (16% and 18% respectively) or recorded no hit within the database (54% and 60% respectively) [17].

Raw sequence data from the cattle pooled sample was mapped to reference genomes of HPV120 and also WUPyV to determine if additional sequence was present that had not been detected in the BLAST search (Table 2). A total of 1433 sequences mapped to the HPV120 reference genome sequence as one contig, covering the minor capsid L2 protein of HPV120 and also a small part of the L1 gene (Table 2). A total of 137 sequences mapped to the WUPyV reference genome in the large T-antigen gene as two separate contigs (Table 2). Sequences for the larger two contigs are available in genbank, accession numbers KF201691 and KF201692. The sequence of these three contigs indicates that they were present in the sequencing library as circular DNA fragments, that are likely to have arisen as an artefact of rolling-circle amplification by the phi29 polymerase used in Illustra GenomiPhi V2 DNA amplification kit.

**Table 2:** Summary of mapping statistics for WU polyomavirus and human papillomavirus 120 sequences identified in the cattle processing area aerosol sample, when mapped to reference genomes GU296398.1 for WUPyV, and JQ963485.1 for HPV120, using the Bowtie 2 software [28].

	WUPyV Assembly		HPV120 Assembly
	Contig 1	Contig 2	Contig 1
Number of reads mapping to reference genome	5	132	1433
Length of contig (bp)	66	349	541
Coverage (min/max)	3X/4X	15X/41X	109X/272X
% identity of contig to reference genome	99.3%	98.8%	98.2%
Mapped gene region & genome position	Large T-antigen	Large T-antigen	L2/L1
Position within reference genome (bp)	3162–3226	4008–4356	4824–5365

**Figure 3:**
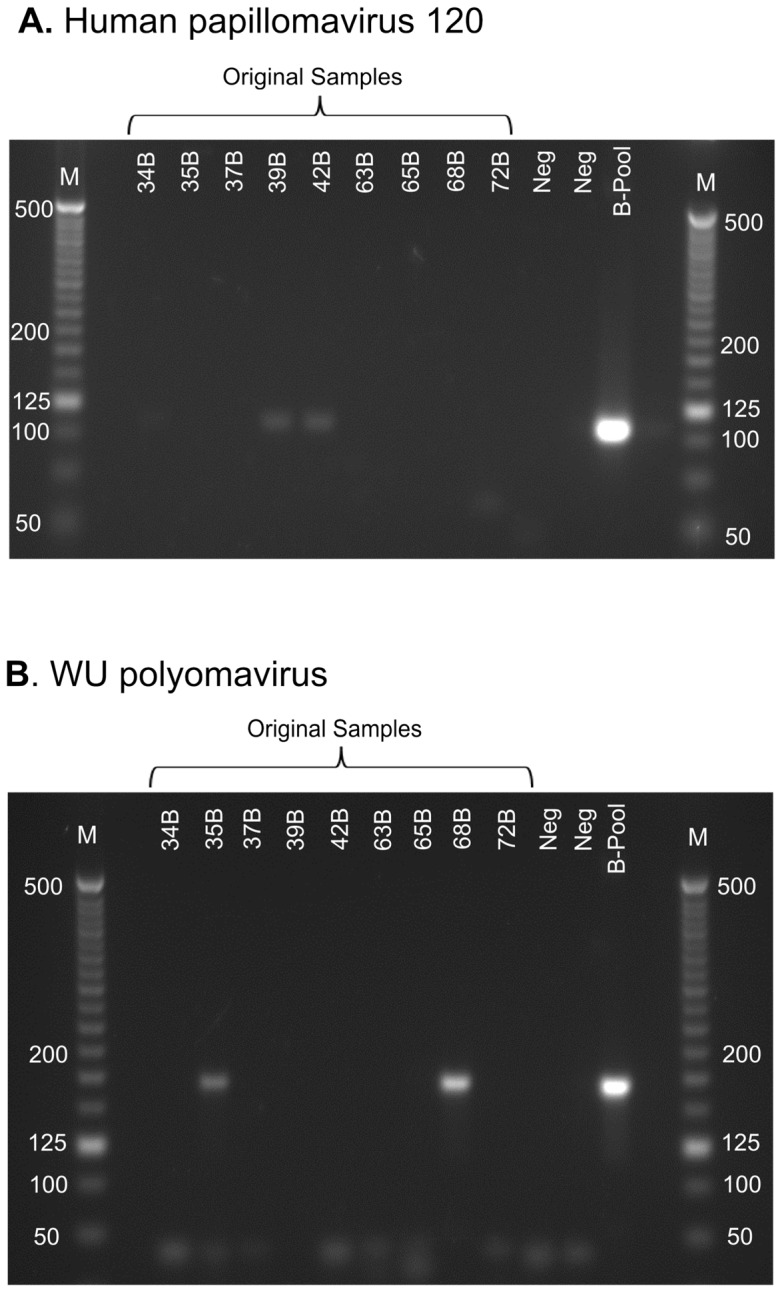
Confirmation of the presence of human papillomavirus 120 (A) and WU polyomavirus (B) sequences in the cattle processing area. Agarose gel electrophoresis (2% w/v) detection of PCR amplicons showing the presence of 160 bp amplicon for WU polyomavirus and 105 bp amplicon for human papillomavirus 120. The pooled-amplified sample (B-Pool) was positive for the presence of both viruses, and results are also shown for DNA extracted from individual personal aerosol samples from each worker (original samples). Negative control samples are included (Neg), alongside a molecular marker (M; gradations in base-pairs).

Specific PCR assays were designed and tested on the pooled samples to confirm that WUPyV and HPV120 sequences were present (Figure 3). The cattle-processing area was positive for both WUPyV and HPV120 amplicons, and subsequent sanger-sequencing of amplicons confirmed both the WUPyV and HPV120 viral sequences (data not shown). The specific PCR assays for WUPyV and HPV120 were also trialled on the original DNA (pre-amplification) from each aerosol sample (Figure 3). The presence of amplicons of the correct size (105 bp for HPV120 and 160 bp for WUPyV) was confirmed in individual aerosol samples from the cattle area, for 2/9 samples tested for WUPyV and 3/9 samples for HPV120 (one weak positive and two strong positives). The individual aerosol samples 35B (meat packaging room) and 68B (red offal collection area) were positive for WUPyV and samples 34B (killing and exsanguination area), 39B (green offal collection area) and 42B (fat trimming area) were positive for HPV120. Neither the HPV120 or WUPyV amplicons were detected in the original DNA samples from the sheep processing area.

## Discussion

To the best of our knowledge, we present the first description of a metagenome derived from personal aerosol samples that is designed to be representative of the inhalable dust/bioaerosol fraction of the air in the workplace environment, specifically in animal slaughterhouses processing cattle and sheep. It was hypothesised that these environments would contain significant amounts of bioaerosol due to the mechanical processes used to kill and process animals, a high degree of splashing and fluid handling, and also the high relative humidity of the environment [29], [30]. Workers in animal slaughterhouses have a high degree of exposure to animal body fluids and tissue, and ‘meatworkers’ in New Zealand are known to have exposure to a number of significant zoonotic diseases including leptospirosis [31]–[33], parapoxvirus ORF (‘scabby mouth disease’ in sheep) [34], and butchers’ warts caused by human papillomavirus subtypes HPV2, HPV4 and HPV7 [35]–[38]. These workers are also affected by a higher-rate of malignancies of the lung compared to the general population, as has been previously described by our group and others [1]–[4], [8], [36]. We sought to describe the metagenome of the bioaerosol within animal slaughterhouses, using an air sampling technology that reflects the personal exposure of meat workers. This work not only provides the basis for a new method for metagenomics, but informs efforts to understand transmission pathways of zoonotic disease in animal slaughterhouses, and provides a snapshot of the environment that may precipitate a higher risk of cancer in this occupational group.

The detection of bovine and ovine sequences was consistent with the environment in which the sampling took place, as random fragments of the bovine and ovine nuclear genome were detected. The BLASTN hits for the Bovinae (cattle) sequences far outnumber the Caprinae (sheep) sequences in the sheep processing area (Figure 2), which is likely an artefact that has occurred due to the greater representation of the cattle (*Bos taurus*) genome in the genbank database as compared to sheep (*Ovis aries*), with 179,286 nucleotide sequence records for *Bos taurus* versus 45,991 nucleotide sequence records for *Ovis aries* (genbank nucleotide core subset; accessed 13 March 2013). The non-uniform representation of different genomes in the genbank database is a factor that is known to affect taxonomic assignment of less well characterised organisms in metagenomic studies [39]. Cattle and sheep are closely related and share high sequence similarity [40] which may further confound this effect. However, it is still apparent that there were 14 times more Caprinae hits in the sheep processing area (24,566 contigs) as compared to the number of Caprinae hits in the cattle processing area (1,758 contigs; Figure 2).

Sequences from bacteria, and also some bacteriophage, were detected in the high-throughput sequencing data in the cattle area (7.4%) and to a lesser extent in the sheep area (0.27%; Figure 2). It is known that bacteriophage can be transferred by aerosolisation, and have been used as a quality control for testing bioaerosol sampling devices [41]. In general, the bacterial species detected were consistent (data not shown) with those anticipated to be present in an environment where pelting and evisceration of animals occur, such as enterococci.

The mechanism by which animal DNA has arrived on the filter cannot be ascertained in the present study, but we suggest it is likely to have been by micro-droplet transfer of either cells or subcellular material containing DNA. Similarly, human DNA was also detected with most sequences being those of the nuclear genome. This finding is not unexpected as the PTFE personal-air sampler is situated close to the nose and mouth of the worker when attached to the lapel of their overalls (located just below the chin, pinned to the upper chest area). It is possible that the sampling system we have chosen may therefore also represent the metagenome of expired air from individual workers. Consistent with this observation, we also report the detection of putative human viral sequences in our metagenomic data. The detection of a human papillomavirus 120 (HPV120) sequence is also not unexpected as human papillomaviruses are known to be an occupational health risk for meatworkers [35]–[38]. Interestingly, some human papillomaviruses are oncogenic [42]. The sequence detected had high identity to HPV120, but lower sequence identity to other human papillomaviruses. This strongly suggests that DNA from HPV120 is present in the sample. This virus has previously been found in a wide-variety of human tissue types including, oral cavity rinse samples [43]–[45], plucked eyebrow hair [46], anal and perianal regions [47], [48], penile warts [47], [49], laryngeal papillomas [50] and vulvar/vaginal lesions [47], [51]. This virus has also been detected in environmental samples of human sewage [52]. It is unknown what involvement HPV120 has in human cancer. It has been shown that human papillomavirus DNA can be disseminated by an aerosol in a plume generated by CO_2_ laser treatment of plantar warts [53], [54] and respiratory tract papillomas [55], but it remains unclear as to whether these virus particles are infectious. We do note that direct contact transfer of HPV120 in our sample is unlikely to have occurred as the filter-apparatus contains the PFTE within an enclosed housing, thus preventing accidental direct contact by the hands of the workers. Human papillomaviruses are already known to be an occupational health risk for meat workers, who contract dermal warts (‘butchers warts’) thought to be caused by HPV2, HPV4 and HPV7 [35]–[38]. It has been suggested that the environmental conditions in animal slaughterhouses may be conducive to the proliferation of these common human viruses [35]–[38]. This may also occur for HPV120, as evidenced by the presence of viral DNA in the bioaerosol. It is not possible to speculate on the involvement this virus may have in tumorigenesis, but given that other HPV are causes of cancer [42], the data we present certainly affirm that this hypothesis deserves attention. Of additional interest was that HPV120 was not observed in samples from the workers in the sheep processing areas. It must also be remembered that the technique of using high-throughput sequencing for this purpose is not validated or standardised, the limit-of-detection has not been ascertained, and this technique is at best a ‘discovery tool’ at present.

The detection of WU polyomavirus (WUPyV) sequence is perhaps also not wholly unexpected. The sequence detected in this metagenomic dataset shows high identity to published WUPyV sequences. WU polyomavirus was initially identified as a potential aetiological agent of respiratory disease in paediatric patients [56]–[58], but polyomaviruses such as JC and BK have also been found to infect healthy control groups at similar rates suggesting human infection is perhaps ubiquitous [59], [60]. Antibodies to WUPyV are present in 89% of healthy adults [61]. WU polyomavirus has also been detected in environmental samples of human sewage [62]. The detection of this virus in our sampling methodology suggests that one of the meatworkers may have had an active infection of WUPyV, that perhaps had remained chronic into adulthood, as described for other polyomaviruses such as JC and BK viruses [56]. Alternately, it could be that we have detected the integration of WUPyV DNA into the human genome of one of the workers. Published literature on WUPyV integration into the host genome does not exist, but must remain a possible explanation for our current findings.

Given the presence of sequences from humans and also their viruses in these samples, this raises the question as to what is the most appropriate way to sample an individual’s microbiological exposure via aerosol. The killing and processing of animals in this occupational setting represents a source for exposure, but there is potential for exhaled human breath from the subject or from other persons present in the area to contribute. Future studies that seek to describe aerosol metagenomes representative of the air inhaled by humans will need to consider this potential and act according to the aim of their study. In our study, our aim was to reveal candidate viral organisms present in the air of animal slaughterhouse, to inform future studies on the possible aetiology of human lung malignancy in workers. We may therefore have been sampling both workplace exposure and workplace infection. Bulk sampling of the aerosol using remote devices is possibly another approach to avoid detection of human exhaled breath, such as in a *de novo* metagenomic study of air-conditioning HEPA filters in shopping centres [13], however, even in this example it was possible to detect commensal bacteria known to colonise humans [13]. Another metagenomic study examined the virome present in outdoor air samples, including a residential site and an industrial complex, and no human viruses were detected [63]. *De novo* metagenomic studies of air are limited to these examples, but these types of methods will be a powerful tool for use in public health studies of the future, such as in high-risk household environments, public transport, and high-risk occupational environments.

Environmental aerosol samples represent a heterogenous collection of a wide variety of organisms, and follow-up studies will require additional sampling regimes, such as swabbing, in order to identify the source material of micro-organisms. In the case of our study, the next step will be to survey meatworkers for the presence of WUPyV (and possibly related polyomaviruses) and HPV120 to confirm that humans are the source of these viral sequences, and undertake full length sequencing of HPV120 and WUPyV to determine the level of conservation across the entire genome with related strains, such as has been described for other papillomaviruses identified in humans [64].

Implicating viruses as an aetiological agent in human cancer is very challenging, particularly when attempting to link exposure via a bioaerosol to the development of cancer, which may not occur until many years after an infection has resolved. This should not deter investigators, but carefully designed large multi-region longitudinal studies will be required to elucidate viral causes of cancer caused by exposure to a bioaerosol. Metagenomic data provides a baseline and starting point for such investigations, including the development of diagnostics and targeted approaches for specific agents. In the event that multiple infectious agents are thought to contribute to increased rates of cancer in meatworkers, then it may be necessary to consider using modified metagenomics Koch’s postulates [65] to determine if metagenomes observed in lower risk areas of the animal slaughterhouse, differ from metagenomes observed in higher risk areas.

This study presents the first metagenomic description of air samples representative of individuals’ exposure in a workplace, and we describe the viral component of a bioaerosol. We present two candidate vertebrate viruses that are worthy of further investigation, HPV120 and WUPyV, given the increased lung malignancies that are observed in the occupational setting of animal slaughterhouses.

## Supporting Information

Figure S1 **Taxonomic assignment of blastn output data for contigs derived from the pooled-aerosol samples in the cattle-processing area.** Assignments to taxa were determined using MEGAN. The size of the circles at each node is proportional to the number of sequence reads assigned, and the exact number of contigs at each node is also shown. The ‘no hits’ category refers to instances where no blastn hit was recorded for a sequence. The ‘not assigned’ category refers to instances where MEGAN could not accurately resolve a taxonomic assignment for a sequence despite blastn hits to sequences in the database occurring.
(TIF)

Figure S2 **Taxonomic assignment of blastn output data for contigs derived from the pooled-aerosol samples in the sheep-processing area.** Assignments to taxa were determined using MEGAN. The size of the circles at each node is proportional to the number of sequence reads assigned, and the exact number of contigs at each node is also shown. The ‘no hits’ category refers to instances where no blastn hit was recorded for a sequence. The ‘not assigned’ category refers to instances where MEGAN could not accurately resolve a taxonomic assignment for a sequence despite blastn hits to sequences in the database occurring.
(TIF)
